# UniFrac – An online tool for comparing microbial community diversity in a phylogenetic context

**DOI:** 10.1186/1471-2105-7-371

**Published:** 2006-08-07

**Authors:** Catherine Lozupone, Micah Hamady, Rob Knight

**Affiliations:** 1 Department of Molecular, Cellular, and Developmental Biology, University of Colorado, Boulder, CO 80309, USA; 2 Department of Computer Science, University of Colorado, Boulder, CO 80309, USA; 3 Department of Chemistry and Biochemistry, University of Colorado, Boulder, CO 80309, USA

## Abstract

**Background:**

Moving beyond pairwise significance tests to compare many microbial communities simultaneously is critical for understanding large-scale trends in microbial ecology and community assembly. Techniques that allow microbial communities to be compared in a phylogenetic context are rapidly gaining acceptance, but the widespread application of these techniques has been hindered by the difficulty of performing the analyses.

**Results:**

We introduce UniFrac, a web application available at , that allows several phylogenetic tests for differences among communities to be easily applied and interpreted. We demonstrate the use of UniFrac to cluster multiple environments, and to test which environments are significantly different. We show that analysis of previously published sequences from the Columbia river, its estuary, and the adjacent coastal ocean using the UniFrac interface provided insights that were not apparent from the initial data analysis, which used other commonly employed techniques to compare the communities.

**Conclusion:**

UniFrac provides easy access to powerful multivariate techniques for comparing microbial communities in a phylogenetic context. We thus expect that it will provide a completely new picture of many microbial interactions and processes in both environmental and medical contexts.

## Background

Sequencing microbial genes directly from the environment has uncovered a vast diversity of microbial lineages that had not been found by techniques that require cultivation [1,2]. The gene encoding the small subunit of ribosomal RNA (SSU rRNA) has been especially useful as a marker for phylogenetic diversity [1]. Although sequences are often used to catalogue the types of microorganisms present in a single environment, comparisons between sequences from multiple environments are increasingly important because they can test whether microbial community composition changes in response to specific environmental variables. Such applications include testing how disease affects oral or gut microbial communities [3-5], understanding industrial processes such as the different types of activated sludge in batch reactors [6], understanding how pollution affects natural ecosystems [7,8], and understanding the basic ecology and distribution of microbes [7,9,10].

When comparing microbial communities, researchers often begin by determining whether each pair of communities is significantly different. However, to gain a broad understanding of how and why communities differ, it is essential to move beyond pairwise significance tests. For example, we might want to know whether differences between communities stem primarily from particular lineages of the phylogenetic tree, or whether there are environmental factors (such as temperature, salinity, or diseases) that group multiple communities together.

Here we introduce UniFrac, a web application that allows researchers to address many of these broader questions about the composition and evolution of bacterial communities. For example, symbiotic communities associated with different animal species would be expected to show differences correlating with the evolutionary distance between the animals if most of the change is through divergence and vertical inheritance. However, if diet or geography influences the assembly of the communities more, we would expect the community differences to correlate more with these other factors. UniFrac uses phylogenetic information and multivariate statistical techniques to determine whether microbial communities are significantly different, identify individual lineages that contribute to these differences, and reveal broad patterns relating many environmental samples. Phylogenetic techniques for comparing microbial communities have only recently been introduced [11-13], and can be more informative than traditional "species"-based methods such as the Jaccard and Sörenson indices (described in [14]). Unlike phylogenetic techniques, species-based methods that measure the distance between communities based solely on the number of shared taxa do not account for the amount of evolutionary divergence between taxa, which can vary widely in diverse microbial populations. Among the first applications of phylogenetic information to comparisons of microbial communities were the Phylogenetic (P) – test and the F_ST _test [12]. These tests were adapted from population genetics, and can be used to determine whether pairs of microbial communities differ significantly. The P-test, in particular, has been broadly applied in microbial ecology [15-20] despite the absence, until recently [21], of a convenient implementation. However, pairwise tests are limited because they can only reveal whether two samples differ from one another, and cannot be used to relate many samples simultaneously.

We recently introduced a new phylogenetic diversity metric called the UniFrac metric, which allows multiple communities to be compared simultaneously [11]. The UniFrac metric measures the distance between communities as the percentage of branch length that leads to descendants from only one of a pair of environments represented in a single phylogenetic tree, i.e. the fraction of evolution that is unique to one of the microbial communities. The metric thus reflects differences between the lineages that are adapted to live specifically in one environment or the others. The UniFrac metric has been used to cluster many different environments according to shared similarities in community composition [3,11]. In this paper, we describe a web application that implements the UniFrac metric and several other phylogenetic tests in a form easily accessible to microbiologists and that does not require any computer programming experience.

The UniFrac web application allows researchers to compare many environments simultaneously using hierarchical clustering and/or principal coordinates analysis (PCA)[22]. The significance of the clusters identified by this method can be established using sequence jackknifing, which tests whether the environments were sampled sufficiently to support individual clusters. UniFrac can also determine whether community differences are concentrated within particular lineages of the phylogenetic tree by applying the G test for goodness of fit [23] to all clades (subtrees) within a tree at a defined distance from the tree root. The interface provides powerful graphics to visualize the differences in PCA plots and phylogenetic trees/environment clusters that are highlighted by significance. Finally, UniFrac can determine whether microbial communities are significantly different using both the UniFrac significance test [11] and the P-test [12], allowing direct comparisons of the two techniques. The UniFrac interface makes the results of these procedures dramatically easier to interpret by providing visual presentations of the data rather than output files that must be plotted in other programs. Thus, UniFrac makes these phylogenetic methods far more broadly accessible to the community of microbial ecologists.

### Implementation

#### Design requirements

Based on user feedback and on our own experience using the original command-line implementation of these analyses, we identified six essential design requirements for the UniFrac system. The system should:

1. Be widely useful and usable by biologists without programming experience.

2. Be fast, scalable, and support multiple simultaneous analyses on a dataset.

3. Support trees from several common phylogeny programs, including ARB [24], PHYLIP [25], PAUP* [26], RAxML [27], MUSCLE [28] and Clustal W [29].

4. Combine a wide range of known and novel phylogenetic algorithms with powerful and easily understood visualization tools. For example, scatterplots of principal coordinates should be able to use either text labels or symbols, and trees should display both branch length and, where available, statistical support.

5. Allow pairwise comparisons between all pairs of environments represented in the input phylogenetic tree to determine which pairs are significantly different after correcting for multiple comparisons.

6. Determine whether community differences are concentrated within particular lineages of the phylogenetic tree.

We were able to meet these design requirements by implementing UniFrac as a web application on a Beowulf cluster (a high-performance network of Linux-based servers). In particular, we achieve scalability by distributing individual components of the analysis to different CPUs in the cluster, which also allows multiple analyses to be performed simultaneously. The web interface also provides an environment familiar to most potential users, and allows the results to be visualized as trees or graphs directly rather than requiring that the data be exported to other programs. Unlike our earlier command-line implementation, the user need not install additional software to run the web version of UniFrac, and the analysis is not limited to a single CPU.

### Data input

UniFrac requires two input files: a single rooted phylogenetic tree that contains sequences derived from at least two different environmental samples, and a file describing which sequences came from which sample. The phylogenetic tree can be in either Newick or Nexus format, and can be generated by programs such as ARB [24], PHYLIP [25], PAUP* [26], RAxML [27], MUSCLE [28] or Clustal W [29]. The environment information must be provided in a text file in which each line contains a sequence name and an environment name separated by a tab. Optionally, the environment file can include a third column describing the number of times the sequence was observed in each environment. This abundance data can come from Restriction Fragment Length Polymorphism (RFLP) data or Operational Taxonomic Unit (OTU) counts.

### Analyses

**Figure 1:**
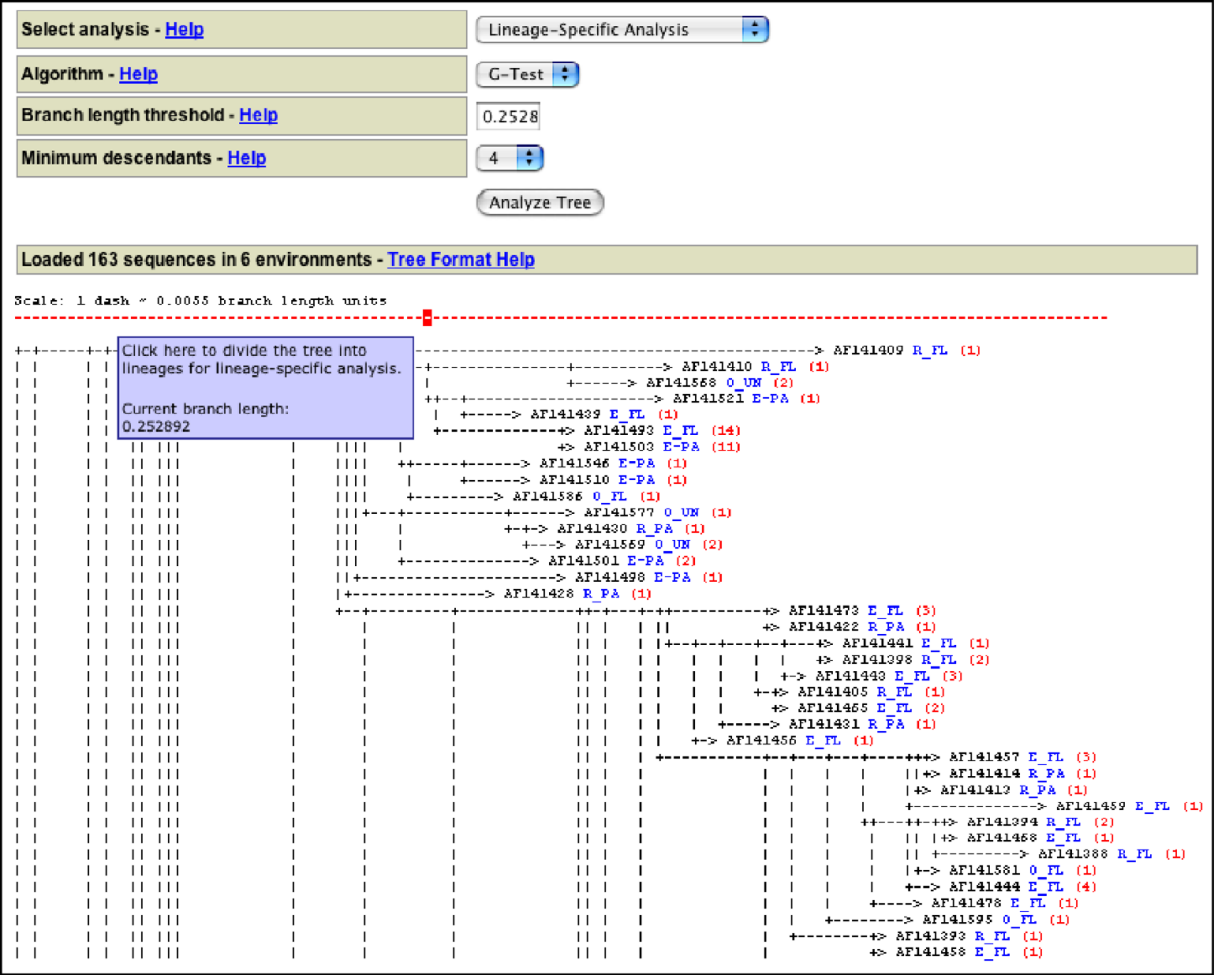
Select analysis page that is displayed after loading a tree and environment file. Only part of the screen is shown with a text representation of the tree. Each branch is labeled with the sequence name in black, the environment in which the sequence was found in blue, and the number of times that it was observed in red. The options for the *Lineage-Specific Analysis *are displayed. The dotted red bar is used to cut the tree into lineages.

**Figure 2:**
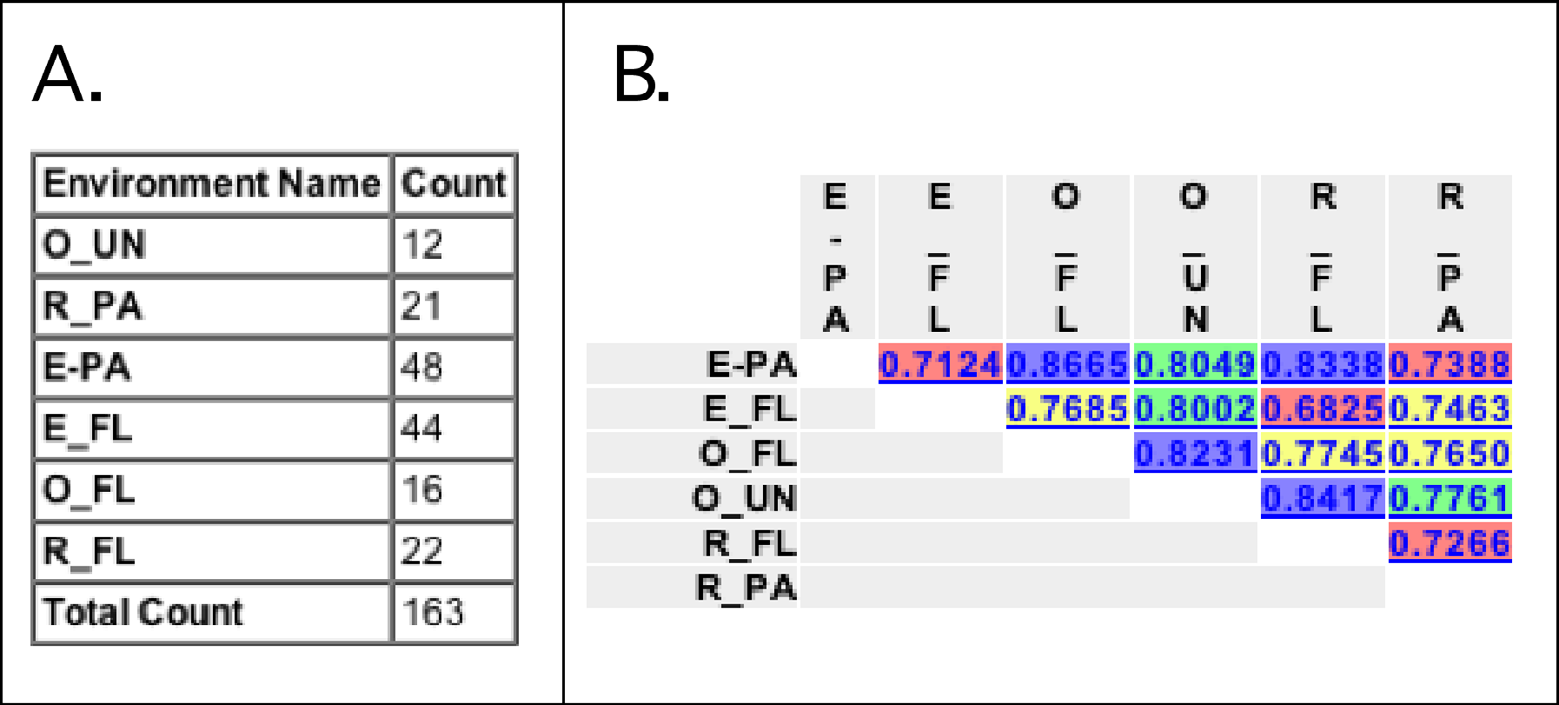
Screenshots of analysis results. For the environment names, the letter before the underscore indicates whether the sequences were from the Columbia River (R), its estuary (E), or the adjacent coastal Ocean (O). The letters after the underscore indicate whether the sequences were from the particle-attached (PA), free-living (FL) bacteria or from unfiltered water (UN). **A**) Result of running the *Environment Counts *Analysis option with *Use abundance weights *set to *No*, so that the counts represent the number of OTUs rather than the total number of clones evaluated (which would sum to 236 instead of 163). **B**) Result of running *Environment Distance Matrix*. The values are colored by quartile; values in the 0–25% range are red, 25–50% are yellow, 50–75% are green, and 75–100% are blue.

UniFrac displays the uploaded data on a page that allows the user to choose among several analyses (Fig. 1). A text representation of the input phylogenetic tree is displayed, including the environments to which each sequence was assigned and the number of times each sequence was observed (Fig. 1). Detailed information on the number of sequences in the tree that were assigned to each different environment can be obtained by selecting *Environment Counts *from the *Select Analysis *drop down menu (Fig. 2A).

### Significance tests

UniFrac allows several different hypotheses about community structure to be tested, using two different methods of calculating significance and three methods for choosing the environments to be compared with one another (Table 1).

**Figure 3:**
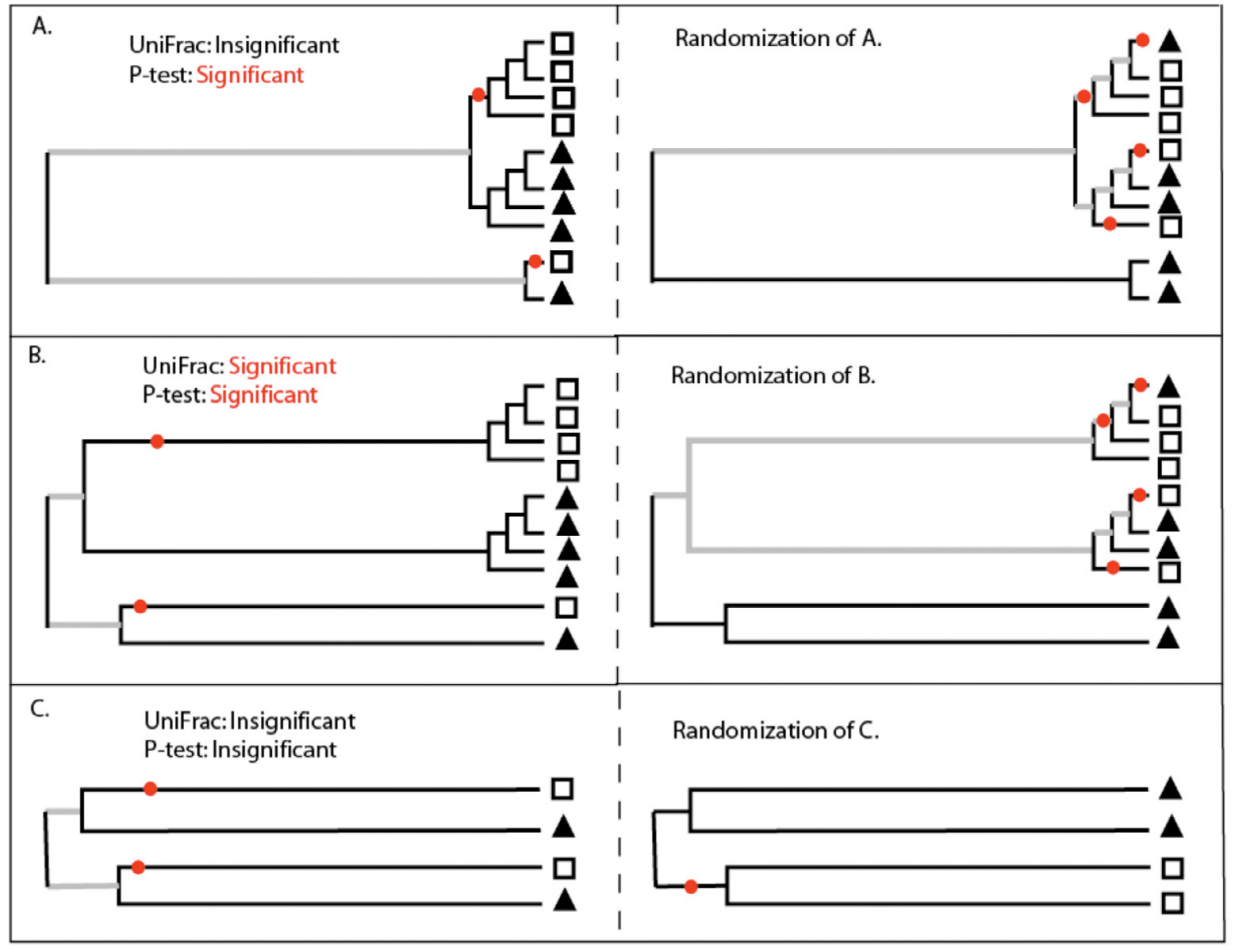
Comparison of the UniFrac Significance test and the P-test with raw and de-replicated data. This figure illustrates how the same tree can have a significant P-test *P*-value and a non-significant UniFrac significance test *P*-value. The trees drawn in A and B have the same topology but different branch lengths. The boxes and triangles represent sequences from two different environments. The trees on the left are being evaluated to determine whether the square and triangle communities are significantly different. The trees on the right are example trees in which the environment assignments have been randomized. The parsimony changes that are calculated with the P-test are represented by red dots. The color of the branches represent calculations made for the UniFrac significance test; branches that lead to only one of the two environments are black and branches that lead to descendants of both environments are grey. **A**.) A tree that would have a significant P-test result and a non-significant UniFrac Significance test result. The sequences from the square and triangle environments are clustered together on the tree, and it thus only takes 2 changes between environments to explain their distribution. This is less than would be expected if the sequences were randomly distributed between environments as shown on the right, and thus the *P*-value is likely to be significant (note that in practice, the true tree is compared to many randomized trees and not just one). The monophyletic lineages occur near the tips of the tree, however, and are not associated with a significant amount of unique branch length (black branches). The UniFrac metric value would thus be low and randomization of the tree could easily result in more unique (black) branch length as shown on right, resulting in a non-significant *P*-value. **B**.) A tree that would have a significant result for both the P-test and the UniFrac significance test. The P-test results are the same as for the tree in A because the topology is the same. However, because the monophyletic lineages in the square and triangle environment represent a substantial amount of branch length in the tree, the UniFrac value is high. The permutations of environment assignments would thus typically result in less unique branch length, leading to a significant result. **C**) The same analysis as B except that the diversity at the tips of the tree has been removed by choosing OTUs. The UniFrac distance is essentially unchanged, but randomization over the reduced number of taxa results in non-significant *P*-values for both the UniFrac Significance test and the P-test.

UniFrac can calculate whether two communities differ significantly using either the UniFrac significance test [11] or the P-test [12]. The P-test estimates similarity between communities using the number of changes from one environment to another along a branch that is required to explain the distribution of sequences between the different environments in the tree (Fig. 3). The *P*-value is the fraction of trials in which the true tree requires fewer changes than trees in which the environment assignments have been randomized. The UniFrac significance test measures similarity between communities as the fraction of branch length in the tree that is unique, meaning that it leads to descendants in one environment or the other but not both (Fig. 3). The *P*-value is the fraction of trials where the true tree has more unique branch length than trees in which the environment assignments have been randomized.

In the UniFrac interface, the *Number of permutations *option selects the number of random permutations of environments used to calculate the *P*-values for the P-test and the UniFrac significance test. Both tests can be performed on *All environments together *or for *Each pair of environments*. Performing the analysis on *All environments together *tests whether the sequences from all of the different environments in the tree are significantly different from each other. For example, if there are sequences from 3 different environments in the tree, only one *P*-value is returned. This *P*-value indicates the probability that the sequences differ significantly across all 3 environments. A significant *P*-value can arise even if only one environment differs from the rest.

In contrast, performing the analysis on *Each pair of environments *returns a *P*-value for each possible pair of environments in the tree. This option can be used to test which pairs of environments differ from one another. Sequences present in neither of the two environments being compared are removed from the tree before calculating the significance. When making many comparisons, *P*-values must be corrected in order to maintain the desired Type I error rate (the probability of incorrectly rejecting the null hypothesis when it is true). We correct for multiple comparisons by multiplying the *P*-values by the number of comparisons that were made, otherwise known as the Bonferroni correction [23], and report both the corrected and uncorrected results of the analysis. This correction can make it difficult to obtain a significant *P*-value for trees with sequences from many different environments, even if the environments have very divergent communities. Pairwise comparisons between more than 3 samples should use at least 1000 randomized trees, because the minimum *P*-value that can be reported for 100 permutations is <= 0.01 (if none of the permuted labels gave a score more extreme than the actual score). Because there are six ways to choose pairs for four samples for comparison, we would need to correct for multiple comparisons by multiplying this minimum value of 0.01 by 6, resulting in a *P*-value of <= 0.06. Thus, no individual pair could be shown to be significantly different at a 0.05 cutoff.

The UniFrac Significance test also has the option to analyze *Each environment individually*, which can determine if a significant *P*-value for *All environments together *is due primarily to a single environment. This could occur if one environment contains only sequences that also appear in the other (analogous to what can be detected using LibShuff [13,30]), or if the sequences in one environment are more tightly clustered or are on longer branches. This analysis returns a *P*-value for each environment, indicating whether that environment has more unique branch length than would be expected if the sequences from that environment were randomly distributed in the tree. The calculations are similar to those for the UniFrac significance test with *All environments together*, except that the fraction of unique branch length is calculated for each environment individually and compared to a random distribution for that particular environment, thus testing whether any single environment differs in phylogenetic distribution from the rest of the sequences overall. The *All environments together *option instead pools the unique branches for all the environments in the tree, thus testing whether any combination of environments is nonrandomly distributed.

Note that whether or not sequences are dereplicated can have substantial effects on the statistical significance of the distance between samples, even though there will be no effect on the distance itself. For example, suppose you have two samples A and B. OTU X appears 10 times in sample A, and does not appear in sample B. After dereplication, only one sequence from X in sample A remains in the data set. During the randomization, in which environment labels are assigned to each taxon independently, the original set (with 10 copies of X) can have each of these copies assigned either to A or to B. Because, on average, half the copies will be found in each environment, in most cases the branch length leading to X will be counted towards both A and B, and so the original data set (which assigns all this branch length to A) will appear to be highly unusual and thus statistically significant. However, in the dereplicated data set, in which there is only one copy of X, all of its branch length will be assigned either to A or to B, but never to both. Consequently, X will always count as "unique branch length", and the real observations will appear no different from randomized observations (see Fig. 3C).

### Lineage-specific analysis

If the communities from two environments are significantly different, the next step is to determine which lineages contribute to the difference. This is commonly done by binning the sequences by taxonomic group (e.g. by division), and then comparing the percentage of sequences from each group in each environment using bar graphs or pie charts. For more detailed analysis, researchers often visually inspect the phylogenetic tree to identify lineages that are overrepresented in one environment, but this is seldom done with statistical rigor. UniFrac's lineage-specific analysis determines whether particular lineages in the phylogenetic tree have an excess or deficit of sequences from a particular environment by applying the G test for goodness of fit to each lineage at a defined distance from the root. The G test is similar to the chi-squared test for goodness of fit, but is more accurate for small sample sizes [23].

To apply the G test, the lineages are first separated by cutting the tree at a specified distance from the root, which can be entered numerically or chosen by clicking on a scale bar above the tree (Fig. 1). Choosing a small distance, which cuts close to the root, can perform a high-level analysis at the division level. Choosing a large distance, which cuts far from the root, can perform a low-level analysis at about the family or genus level. For each lineage, we use the G test to generate a *P*-value that indicates the probability that the actual count of sequences in each environment is significantly different from the counts that would be expected if sequences were randomly distributed in the tree. As with the pairwise significance tests, the *P*-values are multiplied by the number of comparisons that were made (in this case the number of lineages evaluated) in order to maintain the desired Type I error rate [23]. Detecting significant differences is thus difficult when a large distance from the root is specified and many lineages are evaluated.

**Figure 4:**
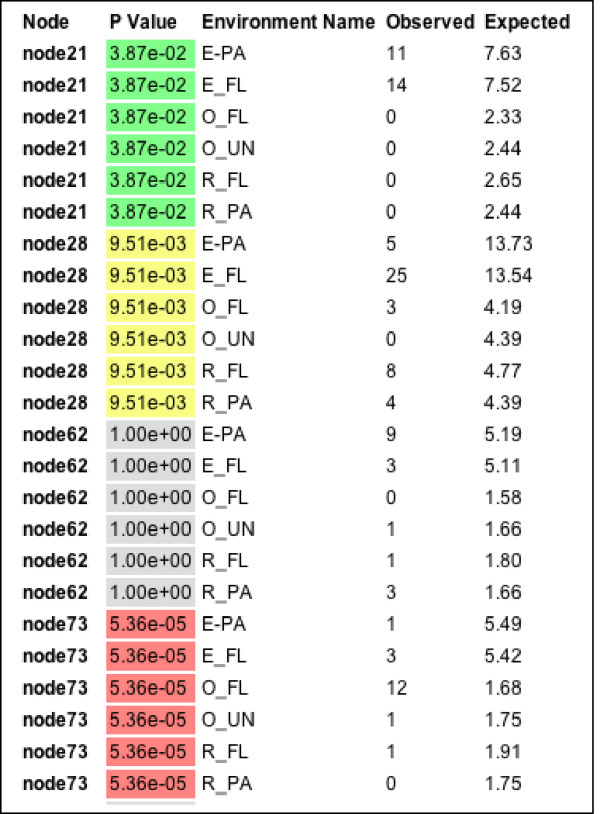
Partial output of the *Lineage-Specific Analysis *with *Minimum descendants *set to 6. The complete output consists of both a table and a tree. The table has a row for each environment in each evaluated lineage/node. The nodes are named arbitrarily but can be viewed in the tree. Each evaluated node is colored based on its *P*-value in both the table and the tree. *P*-values < 0.001 are red, < .01 are yellow, < 0.05 are green, < .1 are blue and > 0.1 are gray. The table shows the observed and expected sequence counts for each environment for each evaluated node. The expected counts are what would be expected if the sequences were evenly distributed in the different lineages.

The lineage-specific analysis can use abundance information provided in the environment file. For instance, if RFLP data indicate that a particular sequence was represented by 15 different clones, the program will count that sequence 15 times when performing the G test. After the lineage-specific analysis, each lineage that was evaluated is colored on the tree according to its level of significance. A table summarizing the *P*-value and expected and observed counts for each environment in each lineage is also provided (Fig. 4).

### Comparing many environments simultaneously

**Figure 5:**
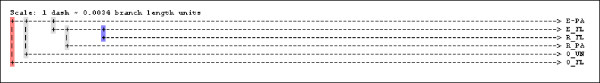
Result of running *Jackknife Environment Clusters *with *Number of sequences to keep *set to 12 and *Number of Permutations *set to 100. The environment abbreviations are the same as described for Fig. 2. Each node is colored by the fraction of times it was recovered in the jackknife replicates. Nodes recovered >99.9% of the time are red, 90–99.9% are yellow, 70–90% are green, 50–70% are blue, and < 50% are grey. The fraction can also be viewed in the interface by moving the pointer over the colored bar.

UniFrac can compare many communities simultaneously using hierarchical clustering with the UPGMA (Unweighted Pair Group Method with Arithmetic Mean) algorithm [25] and PCA (Principal Coordinates Analysis)[22]. We have previously described these techniques and their application in detail [3,11]. Briefly, a distance matrix between environments is made using the UniFrac metric, calculating values for all possible pairs of environments in the tree. This environment distance matrix can be viewed by selecting *Environment Distance Matrix *from the *Select Analysis *menu (Fig. 2B). The environment distance matrix is the input both for clustering with UPGMA, which is executed using the *Cluster Environments *analysis option, and for PCA, which is executed using the *PCA *analysis option. *Cluster Environments *outputs a text representation of a tree in which similar environments will be clustered together (Fig. 5).

The UniFrac interface also has the *Jackknife Environment Clusters *option, which has been described in detail previously [11]. This option indicates how robust each environment cluster is to sample size and evenness by assessing how often it is recovered in randomly chosen sets of sequences (note that this test is performed on groups of environments in the cluster diagram, not monophyletic groups in the phylogeny). To perform this analysis, we randomly sample the same number of sequences from each environment (the *Number of sequences to keep*), re-cluster the environments using these sequences, and calculate the fraction of times that each node in the cluster was recovered. By default, *Number of sequences to keep *is set to the minimum number of sequences found in any environment in the tree. If an environment contains less than the specified number of sequences, all the sequences from that environment are removed, excluding that environment from the cluster output. *Number of Permutations *determines how many random samples are generated (10, 100, or 1000). The output is a tree in which each node is colored according to its jackknife support, i.e. fraction of the random samples that they were recovered in (Fig. 5).

**Figure 6:**
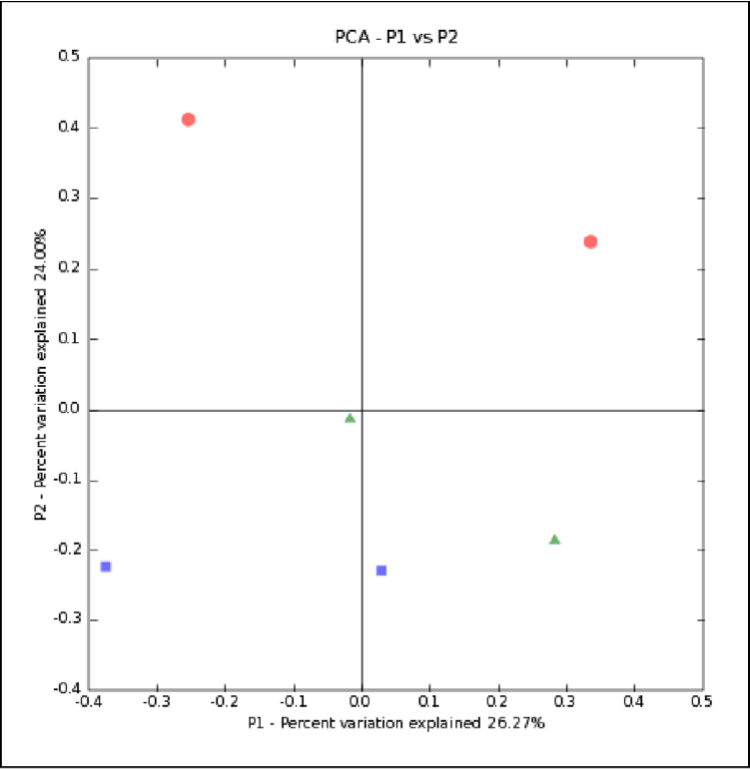
Result of running *PCA *and choosing to output a *ScatterPlot *and the *Bin envs by:first letter *option. Blue squares represent the estuary, green triangles represent the river, and red circles represent the ocean. All points on the left side of the x-axis represent particle-associated bacteria (estuary and river) or bacteria in unfiltered water (ocean). All points on the right side of the x axis are from free-living bacterial communities. The full environment name can be seen by by moving the pointer over the symbols. The axes are labeled with the percent of the variation explained by each principal component.

The environment distance matrix can also be used to cluster the environments using PCA [22]. PCA is a multivariate statistical technique for finding the most important axes along which samples vary. Distances are converted into points in a space with a number of dimensions one less than the number of samples. The principal components, in descending order, describe how much of the variation each of the axes in this new space explains. The first principal component separates out the data as much as possible, the second principal component provides the next most separation, and so forth. The UniFrac interface returns information on all principal component axes in a data table. It also allows easy visualization of that data in scatterplots that compare all pairs of the first three principal components (Fig. 6). The points are either marked with text or colored symbols. Symbols representing environments with similar names are assigned the same color, providing a convenient way to group environments visually by changing the names in the uploaded file. For example, if *Bin envs by: first underscore *is selected, environments that have names containing the same prefix before an underscore will have the same color (for example, "water_A" and "water_B" would get the same color). If *Assign series by: first letter *is selected, environments starting with the same first letter will have the same color (e.g. "soil" and "sediment" would get the same color). Series can also be labeled with the first letter of their names rather than by different colors. PCA often reveals patterns of similarity that are difficult to see on a tree, and the axes along which variation occurs can sometimes be correlated with environmental variables such as temperature or pH. However, when no single principal component explains much of the variance, the clusters generated with *Cluster environments *can be a more useful guide to similarity than the lower-dimensional projections found by PCA.

### Parallelization

Many of the analyses in UniFrac can be parallelized and distributed over multiple CPUs after loading the initial tree and environment file. There are two types of parallelization used in UniFrac: using a single CPU to perform a single analysis (e.g. PCA), and using multiple CPUs to perform a single analysis (e.g. calculating the significance of the differences between all pairs of environments). Depending on the type of analysis requested by the user, one or more CPUs are assigned to handle the request via jobs submitted to the PBS queuing system on our Beowulf cluster. Each analysis request is assigned a unique request identifier, which is then used to track the overall progress of the job or jobs that make up the request. Once all of the jobs are finished running, the partial results are collected from each CPU and are combined before being sent to the user. A user may run several analyses at once (and each request may use multiple CPUs).

## Results and discussion

### Comparing the P-test to the UniFrac significance test

Because the P-test and the UniFrac significance test are evaluating different hypotheses, it is possible to get a significant *P*-value for one and not the other. Fig. 3A describes an example of a tree that would have a significant P-test result and a non-significant UniFrac result. The UniFrac interface allows these two methods to be compared easily for the first time.

The P-test tests the hypothesis that fewer changes from one environment to another are required to explain the actual distribution of modern sequences in environments than would be required if sequences were randomly assigned to environments. A tree in which the sequences are clustered into monophyletic lineages would require few parsimony changes to describe the distribution (Fig. 3). The P-test thus takes the tree topology, but not the branch lengths, into account.

In contrast, the UniFrac significance test accounts for both the tree topology and the branch lengths, and tests the hypothesis that there has been more unique evolution within each environment (more branch length leading to descendants from only one environment) than would be expected if the sequences were randomly distributed among environments. A significant *P*-value thus indicates that the sequences from a particular environment are clustered into monophyletic lineages that represent a longer history of adapting to life in one environment versus the other.

### Usage example

To illustrate the utility of the UniFrac interface, we reanalyzed sequence data generated in a study of bacterial communities in the Columbia River, its estuary, and the adjacent coastal ocean [31]. Crump et al, used filtration techniques to separate particle-attached and free-living bacteria from the three sites, and analyzed these populations separately. Because the particle-attached bacteria in the estuary are 10–100 times more active than free-living bacteria, and because particles have a much longer residence time in the estuary than water (2 to 4 weeks verses 1 to 2 days), Crump at al. hypothesized that the estuarial particle-attached but not free-living bacteria would form a unique community that is distinct from the river and coastal ocean source communities.

In order to test this hypothesis, Crump *et al*. partially sequenced 215 16S rRNA clones from the particle-attached and free-living bacteria in the river, estuary, and coastal ocean, and characterized an additional 24 clones from the estuary using RFLP band patterns. We downloaded the sequences from GenBank, and used the author's annotations to assign each sequence to one of the 6 environments. RFLP screening prior to sequencing was carried out only for the estuary. Whether or not sequences are de-replicated can have substantial effects on the statistical significance of UniFrac distances and the P-test, but only a minimal effect on the UniFrac distances, so the *PCA *and *Cluster Environments *(without jackknifing) analysis results will be unchanged (see Fig. 3C). In order to make the sequence collections comparable between the samples, we de-replicated the sequence data from each of the 6 environments individually by picking OTUs at 97% sequence identity using the "Percent Sequence Identity with gaps" algorithm of FastGroupII [32]. Alternatively, we could have used the RFLP counts to produce a tree in which the same sequence was repeated multiple times, once for each RFLP count. This approach better accounts for the abundance of particular types when assessing significance between samples. (Fig. 3C). For the de-replicated tree, we created an environment file that included only one representative of each OTU, along with the number of times that each OTU was observed. We aligned the sequences using the NAST-alignment tool [33], and used the parsimony insertion algorithm of the Arb package [24] to create a single phylogenetic tree that contained all of the sequences. We exported this tree in Newick format for input into UniFrac.

We successfully assigned environment information to all but 3 of the 239 clones using the authors' annotations. After loading the tree and environment information into the UniFrac interface, we selected the *Environment Counts *analysis option to get detailed information on how many OTUs from each environment are in the study (Fig. 2A). This indicated that more OTUs from the estuary were evaluated than from the river or coastal ocean, and that screening for OTUs reduced the number of sequences in the tree from 215 to 163. UniFrac automatically removes sequences in the input tree that are not assigned to an environment before any analyses are performed.

In order to get an initial idea of how the 6 environments related to one another, we calculated raw UniFrac values for all pairs of environments with the *Environment Distance Matrix *analysis option (Fig. 2B). Small UniFrac values indicated communities that were more similar. A quick glance at the distance matrix, for instance, showed that the free-living bacteria in the Estuary (E-FL) were more similar to the free-living bacteria in the river (R-FL; UniFrac value = 0.6825) than in the ocean (O-FL; UniFrac Value = 0.7685).

In order to better visualize the overall patterns of variation, we used this distance matrix to perform PCA by selecting the *PCA *analysis option. We produced a scatterplot of the first two principal coordinates and colored the points by environment (ocean, river, or estuary) by selecting the *ScatterPlot *and *Assign series by: first letter *option (Fig. 6). PC1 and PC2 explained almost equal amounts of the variation in the data (26.27 and 24.00% of the variation respectively). PC1 separated free-living bacterial communities from particle-attached communities (Fig. 6). PC2 separated the environments from the estuary, river, or ocean; Bacterial lineages in the estuary were more similar to those in the river than to those in the ocean.

We also used the distance matrix to cluster the environments using UPGMA (Fig. 5). At the same time, we determined the robustness of the results to sampling effort and evenness using the *Jackknife Environment Clusters *analysis option with *Number of sequences to keep *set to the default value of 12 and *Number of Permutations *set to 100. We set *Number of sequences to keep *to 12 because this was the number of sequences in the environment represented by the fewest OTUs (O_UN) (Fig. 2A). If we had picked a number greater than 12, all sequences from O_UN would have been removed from the tree and O_UN would have been excluded from the analysis.

Like the PCA results, the UPGMA results suggested that the estuary and river sequences from both the particle-attached and free-living bacteria were more similar to each other than they were to sequences from the ocean, since they clustered together to the exclusion of the ocean samples (Fig. 5). When only 12 OTUs from each environment were considered, however, only the node that grouped the free-living bacteria in the estuary and the river together was supported > 50% of the time (Fig. 5). This lack of bootstrap support indicated that more sampling would be required for confidence in the results for the other nodes.

**Figure 7:**
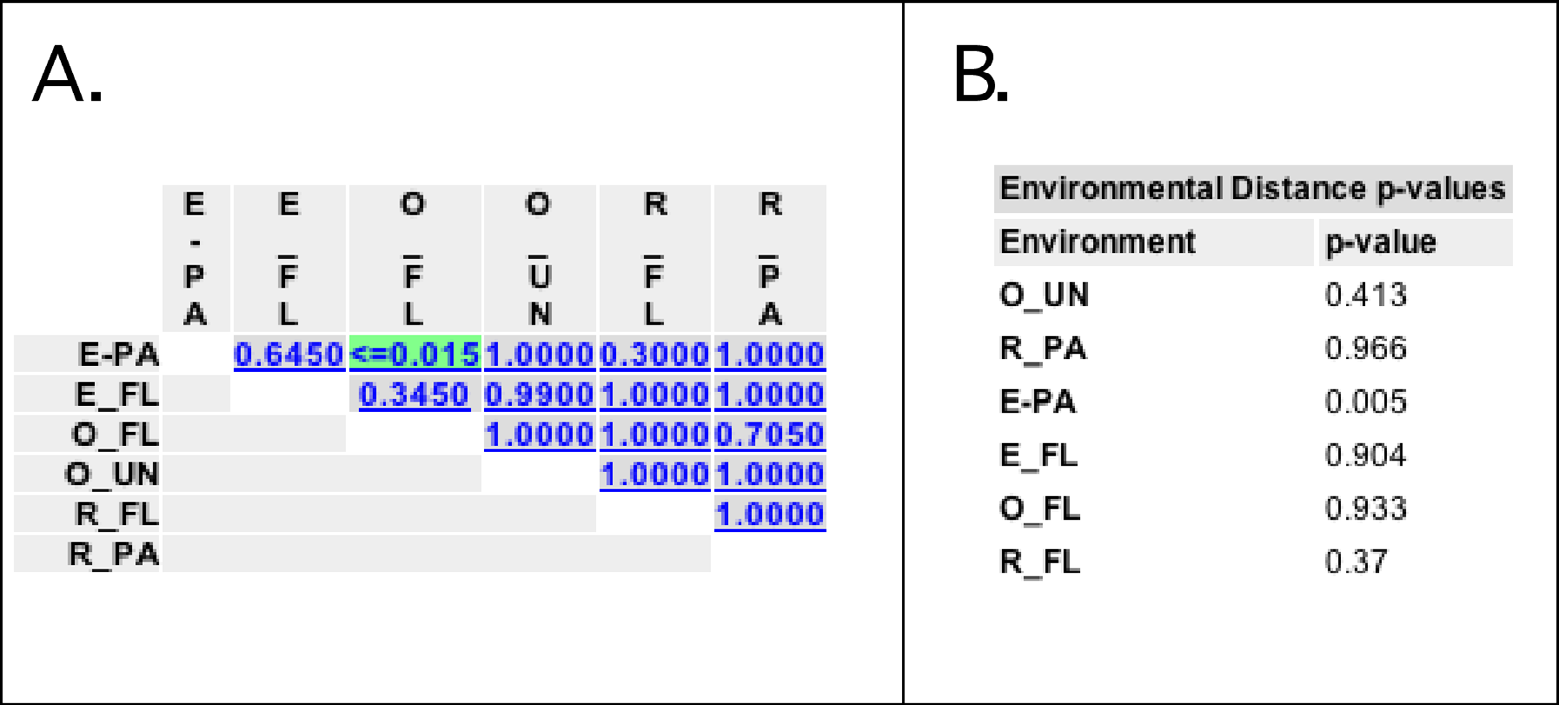
Screenshots of selected significance test results. Environment abbreviations are the same as described for Fig. 2. **A**) Result of running *P-Test Significance *with the *Each pair of environments option*. The *P*-values have been colored by significance. *P*-values < 0.001 are red, 0.001–0.01 are yellow, 0.01–0.05 are green and 0.05–0.1 are blue and >0.1 are grey. **B**) Result of running *UniFrac Significance *on *Each environment individually *with *Number of Permutations *set to 1000.

We next used the *UniFrac Significance *and *P Test Significance *Analysis options to determine whether the communities in the different samples were significantly different from each other. Performing *UniFrac Significance *and *P Test Significance *on *All environments together *and *Number of Permutations *set to 1000 resulted in a non-significant *P*-value for UniFrac Significance (*P *= 0.648) and a significant *P*-value for the P-test (*P *= 0.006). This result indicates that the sequences were significantly clustered by environment overall, but that these clusters did not represent a significant amount of unique branch length (see Fig. 3). Performing *UniFrac Significance *and *P Test Significance *on *Each pair of Environments *with *Number of Permutations *set to 1000 resulted in a significant P-value only for the comparison of free-living bacteria in the ocean (O_FL) and particle-associated bacteria in the estuary (E_PA) for both tests after correcting for multiple comparisons. The level of significance was much stronger with the P-test (*P *< 0.015, Fig. 7A) than the UniFrac Significance test (*P *= 0.030). To correct for multiple comparisons, all of the pairwise *P*-values have been multiplied by 15, because that is the number of comparisons that were made. In the case of the O_FL, E_PA comparison, the *P*-value of < 0.015 indicates that a lower number of parsimony changes was never observed in the 1000 random permutations that were performed.

In order to determine if any particular environment was associated by more unique branch length than expected by chance, we performed *UniFrac Significance *on *Each environment individually *(Fig. 7B). Of the 6 environments, only the particle-associated bacteria from the estuary (E_PA) had a significant difference (*P *= 0.005). This result indicated that the sequences from this sample are associated with more unique branch length than would be expected if they were randomly distributed in the tree, suggesting that bacteria adapted to this environment seldom transfer to and survive in other environments (and vice versa).

Finally, in order to identify lineages that are contributing to differences between environments, we performed a *Lineage-Specific Analysis *with a *Branch length threshold *of 0.252892 and *Minimum Descendants *set to 6. The branch length threshold was chosen using the scale bar (Fig. 1) to separate the tree into lineages that were at or slightly below the division level. The split divided the tree into nine nodes that each had at least six descendants (specified with the *Minimum Descendants *option). Of these, four (nodes 21, 28, 73, and 146) had significant G test *P*-values. Significant values indicated that the node had an excess or deficit of sequences in particular environments relative to chance expectations. The part of the table that describes the results for three of the four significant nodes is shown in Fig. 4. By examining its descendant sequences, we determined that Node 28, which had a G test *P*-value of 0.00951, represented the β-proteobacteria. Comparing the observed and expected counts demonstrated that there were more β-proteobacterial sequences in the free-living components of the estuary and river than expected, and fewer in the other environments (Fig. 4). Node 21 had a G test *P*-value of 0.00387, consisted of members of the Oceanospirillum group of the γ-Proteobacteria, and only had sequences from the estuary. Node 73 had a G test *P*-value of 0.0000536, included more free-living ocean bacteria than expected by chance, and corresponded to the SAR11 cluster within the α-proteobacteria. The rest of the α-proteobacteria were represented by Node 62, and do not show non-random distribution by the G test. The final significant node, Node 146, was overrepresented in the ocean samples and represented the cyanobacteria.

The analysis of these data using the UniFrac interface provided insights that were not apparent from the initial data analysis by Crump *et al*. [31], which used other commonly employed techniques. These included 1) evaluating the identity of the top blast hit for each sequence in each environment, 2) comparing pie charts describing the distribution of sequences between division and subdivision groups in the different environments 3) visually inspecting phylogenetic trees to qualitatively determine whether sequences from the same environment clustered together, and 4) estimating the percentage of sequences in the estuary samples that clustered closely with river or ocean source communities in the phylogenetic trees. Using these techniques, Crump *et al*. concluded that the particle-attached bacteria form a uniquely adapted estuarine community, since 75% of the particle-attached clones in the estuary were rare or absent in the river or ocean (at a cutoff of ~0.96 sequence identity). In contrast, they concluded that the free-living estuarine community was more of a sum of its source communities since about half of the free-living bacteria were similar to clones in the river or coastal ocean.

One conclusion that can be drawn from the UniFrac analysis that was not apparent in the initial analysis is that the estuarine bacteria are more similar to the river bacteria than to the ocean bacteria, both for the particle-attached and free-living fractions. These relationships can be seen in both the UPGMA cluster and the PCA plot. Because UniFrac considers phylogenetic lineages and not just shared OTUs, the results could not stem from the simple explanation that more individual species have the ability to live in both the river and the estuary. Instead, they are consistent with the hypothesis that freshwater bacteria can more easily adapt to the estuarine environment than can ocean bacteria.

As suggested by Crump *et al*., there is some evidence that the estuarine free-living bacteria are more similar to the source communities than are the particle-attached bacteria. However, the UniFrac analysis shows that this evidence is limited to the river source. For instance, the estuarine and riverine free-living bacteria (E_FL and R_FL) formed the only jackknife-supported node in the UPGMA cluster, clustering more closely together than the estuarine and riverine particle-attached bacteria (E_PA and R_PA). The oceanic bacteria, however, did not cluster with either the free-living or the particle-attached estuarine bacteria. The results of the *UniFrac Significance test *on *Each environment individually *also support the hypothesis that the estuarine particle-attached bacteria, in particular, form a uniquely adapted community. Only this environment resulted in a significant *P*-value when compared against the rest of the tree, indicating that it has significantly more unique branch length than the other environments.

The *Lineage-Specific analysis *identified many of the same lineages that Crump *et al*. described as being important for a particular environment. These lineages include the SAR11 cluster for free-living marine bacteria, the β-Proteobacteria for free-living freshwater bacteria, and the Oceanospirillum group of the γ-Proteobacteria for the estuary. Notably, the G test returned a non-significant *P*-value (*P *= 1.0 after correcting for multiple comparisons) for the Cytophaga-Flexibacter group, a group that Crump *et al*. concluded to be particularly important for the estuary. Although the estuary sequences are abundant in this lineage, one would expect them to be abundant in any particular environment by chance because about three times as many sequences were sampled from the estuary as from the other environments. There was thus no statistical support for overrepresentation of estuarine sequences in this lineage.

## Conclusion

Phylogenetic comparisons among communities, especially those that allow the simultaneous comparison of many different communities, have until now been unavailable to research groups that lack programming expertise. By providing a convenient web interface, UniFrac paves the way for a far broader application of these techniques in microbial ecology. In particular, the ability to cluster many communities according to the lineages they contain without needing to choose arbitrary OTUs will greatly enhance our ability to understand the factors that underlie similarities and differences in microbial community samples (we only de-replicated the Crump *et al*. data because RFLPs had been used to select clones for sequencing from some, but not all, samples, and because we were doing significance tests as well as clustering).

The UniFrac web interface contains many enhancements over the command-line implementation of the UniFrac metric we released last year [11]. These enhancements include additional functionality, in particular the taxon jackknifing and the lineage-specific analysis, improved accessibility, powerful visualization tools, and improvements in run-time both through parallelization and through code optimizations. In particular, optimizing certain tree handling routines improved the speed of some tests by a factor of 100 (data not shown). The new visualization techniques, in particular, will greatly assist in exploratory analyses by providing the output in a form that can quickly be interpreted by researchers.

This web interface also provides the ability to compare significance tests based on the UniFrac metric and the P-test within a single interface. Future extensions will include the addition of other significance tests for comparing communities, such as those implemented by PhyloCom [34]. The availability of different tests in the same convenient interface will greatly assist the ability of researchers to compare the plausibility of different hypotheses about community structure. We thus expect UniFrac to usher in a new era of community comparison studies, in which hypotheses about the factors underlying similarities and differences in multiple environments can be conveniently tested, and in which the lineages responsible can be rapidly unmasked.

## Availability and requirements

Project name: UniFrac

Project home page: 

Operating system(s): Platform independent

Programming language: Python

Licence: GPL

Any restrictions to use by non-academics: None

## Authors' contributions

CL drafted most of the manuscript and the online help, wrote most of the code for the calculations, and aided in interface design. MH built the web interface, parallelized the code and improved the performance of the calculations, and contributed to other parts of the code. RK conceived of and coordinated the project, drafted parts of the manuscript and created the tutorial. All authors read and approved the final manuscript.

## Figures and Tables

**Table 1:** Different options in UniFrac allow different hypotheses to be tested.

Option	Hypothesis
P-test	There have been fewer transfers of lineages between environments than would be expected by chance, as measured by counting the number of transfers in a sample of trees in which sequences are randomly assigned to environments.
UniFrac	There is less evolutionary history shared between a pair of environments than would be expected by chance, as measured by adding up the unique branch length (that leads to only one particular environment) in a sample of trees in which sequences are randomly assigned to environments.

Each environment individually	When each environment is compared against all other environments taken together (i.e. all other environments are given the same label), the sequences from that environment alone are nonrandomly distributed on the tree when only changes to/from that environment are considered.
Each pair of environments	When each environment is compared against each otherenvironment (removing all other environments except that pair from the tree), changes between that pair of environments occur less frequently than would be expected by chance.
All environments together	When all environments are included in the same comparison, the total number of changes between environments is less than would be expected by chance, counting any change between different environments as a change.
